# Intelligent monitoring system for pipeline status based on MEMS acoustic emission sensors

**DOI:** 10.1038/s41378-026-01367-1

**Published:** 2026-07-09

**Authors:** Tao Liu, Wenwu Yan, Dongxiao Li, Jidong Cai, Jian Feng, Hanjie Dou, Jiaqian Yang, Pengfan Wu, Hongliang Wang, Xiaojing Mu

**Affiliations:** 1 State key Laboratory of Extreme Environment Optoelectronic Dynamic Measurement Technology and Instrument, Science and Technology on Electronic Test and Measurement Laboratory, North University of China, Taiyuan, PR China; 2 Key Laboratory of Optoelectronic Technology & Systems Ministry of Education, International R & D center of Micro-nano Systems and New Materials Technology, Chongqing University, Chongqing, PR China

**Keywords:** Electrical and electronic engineering, Physics

## Abstract

Pipeline structural health monitoring is critical for global energy security, yet traditional bulk piezoelectric acoustic emission (AE) sensors are inherently bulky, narrow-band, and unsuitable for large-scale, distributed deployment. Here, we present a highly integrated, broadband microelectromechanical systems (MEMS) AE sensor based on ScAlN piezoelectric micromachined ultrasonic transducers (PMUTs) for intelligent pipeline damage monitoring. To address the acoustic impedance mismatch between the silicon-based micro-chip and external media, a composite acoustic matching layer comprising epoxy resin doped with 60 wt% Al_2_O_3_ powder was engineered. This packaging strategy significantly enhances acoustic transmission efficiency while ensuring exceptional mechanical robustness in harsh environments. Systematic characterizations reveal that the fabricated MEMS AE sensor exhibits a broad bandwidth with displacement sensitivity exceeding 60 dB across 40–600 kHz, and a peak sensitivity of 88.4 dB at 335 kHz. In standard pencil lead break tests, it demonstrates a signal amplitude approximately twice that of commercial AE sensors. Furthermore, the device maintains stable performance under severe thermal shock cycling from −55 °C to 85 °C. By integrating this hardware with a Short-Time Fourier Transform (STFT) and a Residual Neural Network (ResNet)-based deep learning algorithm, we developed an intelligent pipeline monitoring system. The system successfully captured and classified the time-frequency characteristics of five distinct human-induced destructive behaviors with an outstanding recognition accuracy of 100%. This work provides a scalable, high-performance hardware-software paradigm for distributed structural health monitoring in extreme industrial environments.

## Introduction

As foundational raw materials of the modern industrial system, oil and natural gas are utilized across a broad spectrum of sectors, ranging from energy generation to chemical manufacturing. Their widespread application is fundamentally driven by their high energy density and ease of extraction and transportation, perfectly aligning with the sustained global demand for efficient energy sources since the Industrial Revolution^1^. According to data from the International Energy Agency (IEA), oil and natural gas account for more than 50% of the global primary energy consumption mix as of 2024. Pipelines serve as the critical infrastructure of the global energy distribution network. Functioning as the vital artery connecting energy production sites to consumer markets, they play an indispensable role in ensuring supply stability, minimizing transportation costs, and enhancing operational safety^2,3^. The geographic mismatch between global petroleum reserves and major consumption markets, coupled with complex topographical challenges such as mountains, deserts, and permafrost regions, constitutes the primary constraint on pipeline routing. Consequently, over 60% of global oil transmission pipelines must traverse remote, unpopulated, and hostile terrains, including wildernesses, arid deserts, and primeval forests^4^. The extreme environmental conditions and highly restricted operational windows in these remote regions pose severe challenges to traditional, manual-based pipeline inspection and maintenance. In contrast to long-haul oil pipelines, natural gas distribution networks are densely integrated within urban environments, typically requiring high internal operating pressures to maintain transmission efficiency^5^. However, urban natural gas pipelines are highly susceptible to leakage risks caused by external damage (e.g., third-party construction excavations and mechanical impacts) as well as structural aging over prolonged service periods^6^. In the event of a rupture or leak, the release of highly flammable and explosive gases poses an immediate and severe threat to public safety and local ecosystems^7,8^. Therefore, the implementation of robust structural health monitoring (SHM) systems for pipeline networks is of paramount importance. Such systems are essential not only for the real-time detection of structural defects and human-induced sabotage but also for guiding cost-effective, predictive maintenance and replacement strategies.

Variations in the structural integrity of pipelines are typically accompanied by fluctuations in physical parameters, including vibration, acoustics, internal pressure, flow rate, and temperature, which serve as crucial indicators for developing highly efficient monitoring systems^9^. Driven by continuous advancements in sensing modalities, microelectronics, and data analytics, various pipeline inspection instruments have been successfully developed and commercialized. Nevertheless, these conventional techniques exhibit distinct limitations: Although accelerometers are easy to install and can capture leak-induced vibrations externally, their applicability for long-distance monitoring is severely hindered by the significant attenuation of high-frequency signals^10,11^. Submersible hydrophones, deployed within the pipeline fluid to localize leaks via acoustic waves, offer a cost-effective solution for small-to-medium, low-noise liquid pipelines; however, they suffer from inadequate anti-interference capabilities, restricted detection ranges, and poor adaptability to complex operating conditions^12,13^. Pressure sensors can identify leaks by monitoring internal pressure transients but generally exhibit sluggish dynamic responses and limited sensitivity to incipient or minor leaks^14–16^. Fiber-optic sensors feature rapid responses to vibration, temperature, and strain with high detection sensitivity; yet, the necessity for long-distance optical cable deployment renders the overall system prohibitively expensive^17–20^.

In contrast, the emergence of acoustic emission (AE) sensors offers a highly promising paradigm to overcome these technological bottlenecks^21,22^. AE is fundamentally defined as the generation of transient elastic waves resulting from the rapid release of strain energy within a material. Spanning a broad frequency spectrum (from several kilohertz to megahertz), and given that even minute structural defects can trigger pronounced AE activities, this technology inherently possesses the advantages of dynamic, in-service, and highly sensitive monitoring, coupled with ease of installation^23,24^. Through advanced data analytics and pattern recognition of AE signals, it is possible not only to accurately perceive pipeline anomalies but also to extract critical geometric and spatial information, such as the location, size, and morphology of cracks, as well as leak dimensions. Consequently, AE has been proven to be a superior technological scheme for detecting structural defects, mechanical damage, and leakage in pipelines^25,26^. However, the AE sensors predominantly utilized in current industrial applications still rely on bulk piezoelectric ceramics as their core transducing elements. These conventional bulk AE sensors are plagued by inherent drawbacks, including bulky dimensions, heavy mass, low integration density, poor device-to-device consistency, and insufficient frequency response flatness^27,28^. Consequently, they fail to accommodate the stringent spatial constraints of compact microsystems and are fundamentally inadequate for the low-cost, dense, and distributed multi-point deployment required by modern structural health monitoring networks.

Driven by the rapid advancements in micro/nanofabrication and advanced electronics in recent years, microelectromechanical systems (MEMS) technology has emerged as a critical pathway to overcome the miniaturization and integration bottlenecks of conventional sensors^29,30^. This shift leverages the inherent advantages of MEMS, including ultra-compact size, high integration density, cost-effectiveness, and functional scalability^31^. For instance, Feng et al. pioneered the development of piezoelectric MEMS AE sensors utilizing multilayer polyvinylidene fluoride (PVDF) as the core sensing element^32,33^. By employing a parallel electrical configuration across multiple piezoelectric layers, this design effectively enhanced the charge output and signal-to-noise ratio (SNR) of the sensor. However, this architecture also exhibits notable limitations, primarily its inferior sensitivity flatness across the operational frequency band, which intrinsically restricts its capability to stably detect AE signals across varying frequencies. Addressing the demand for broadband AE detection, Butaud et al. proposed a design scheme based on multi-frequency capacitive micromachined ultrasonic transducers (CMUTs)^34^. This approach successfully achieved AE signal acquisition over a wide frequency range, offering a novel perspective to overcome the narrow bandwidth constraints of traditional sensors. Nevertheless, the relatively low SNR of CMUTs impedes the precise identification of weak AE signals. Furthermore, the requirement for a high DC bias voltage, combined with specialized packaging constraints, exposes these sensors to severe challenges concerning long-term stability and environmental adaptability in harsh industrial scenarios, such as high temperatures, intense vibrations, and elevated humidity, thereby impeding their widespread industrial deployment. Additionally, researchers have explored various alternative structural configurations for MEMS AE sensors, including integrations with piezoresistive elements, accelerometers, or nanofiber composite membranes, and have conducted preliminary application tests in structural damage detection scenarios^35–37^. However, these studies have predominantly remained at the proof-of-concept functional demonstration level. They often lack systematic calibration of core performance metrics, such as absolute sensitivity, bandwidth, frequency flatness, and operational reliability, resulting in ill-defined performance boundaries that struggle to meet the stringent precision and stability requirements of industrial inspections^38–40^. Therefore, there is an urgent need to develop a higher-performance, highly reliable MEMS AE sensor. Such an advancement is of profound significance for broadening the application spectrum and driving the technological evolution of industrial pipeline monitoring.

In this work, we present a highly integrated, high-performance MEMS AE sensor and validate its application in pipeline sabotage monitoring, fully demonstrating its practical feasibility for industrial scenarios. The proposed MEMS AE sensor consists of piezoelectric micromachined ultrasonic transducers (PMUTs), a signal conditioning circuit, epoxy resin, Al₂O₃ powder, and a metal housing. Serving as the core sensing elements of the system, the PMUTs offer the advantages of compact size, high sensitivity, and batch fabricability. Furthermore, we introduce a dedicated encapsulation method using epoxy resin doped with 60 wt% Al₂O₃ powder to achieve efficient acoustic impedance matching; this compact device integration also enhances the sensor’s reliability in extreme environments. Pencil Lead Break (PLB) tests demonstrate that the proposed MEMS AE sensor exhibits a sensitivity approximately twice that of conventional bulk ceramic AE sensors. Additionally, performance characterization via the comparison method confirms that the sensor maintains a sensitivity exceeding 60 dB [ref V/(m/s)] across the 40 kHz–600 kHz frequency band, with a peak sensitivity of 90.4 dB at 310 kHz. To further validate the sensor performance, a simulated pipeline sabotage monitoring system was developed. Utilizing a Residual Neural Network (ResNet), this pipeline health monitoring system achieved an extraordinary 100% accuracy in identifying five distinct pipeline sabotage behaviors, demonstrating the tremendous promise of the proposed MEMS AE sensor for widespread deployment in industrial pipeline SHM.

## Materials and methods

### Principles of pipeline condition monitoring

Pipelines serve as the critical arteries for the long-distance transportation of oil and natural gas, as well as the essential infrastructure for urban household energy distribution. Third-party sabotage targeting pipelines not only incurs direct energy losses but also inflicts severe ecological damage. Concurrently, structural degradation induced by harsh natural environments, corrosive internal media, and material aging poses a direct threat to public safety and social stability. Consequently, high-performance MEMS AE sensors are of paramount importance in the realm of pipeline condition monitoring, as their sensing capabilities directly dictate the accuracy of fault detection and localization.

**Fig. 1:**
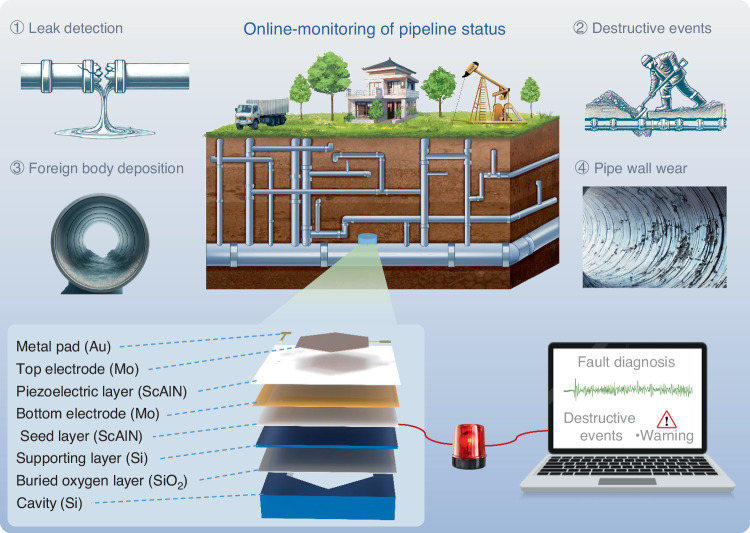
Online-monitoring system for pipeline status based on MEMS AE sensor

As illustrated in Fig. 1, the MEMS AE sensors are installed on the exterior surface of the pipeline to acquire AE signals carrying distinct characteristics. These signals are typically triggered by pipeline leakages, human-induced sabotage, foreign object deposition, and pipe wall wear, thereby facilitating real-time, online monitoring of the pipeline’s operational status. The exploded view reveals the architectural composition of the PMUTs, the core sensing element of the MEMS AE sensor, which primarily comprises a top electrode, a piezoelectric layer, a bottom electrode, a support layer, and a released cavity. During operation, both the intrinsic acoustic signals generated by the medium transmission and the transient elastic stress waves induced by leakages or destructive events are captured by the PMUT and transmitted to the processing terminal. Ultimately, coupled with a ResNet for signal classification and pattern recognition, the system realizes intelligent, online diagnostics of the pipeline’s structural integrity.

### Design and fabrication of the PMUTs chip

**Fig. 2:**
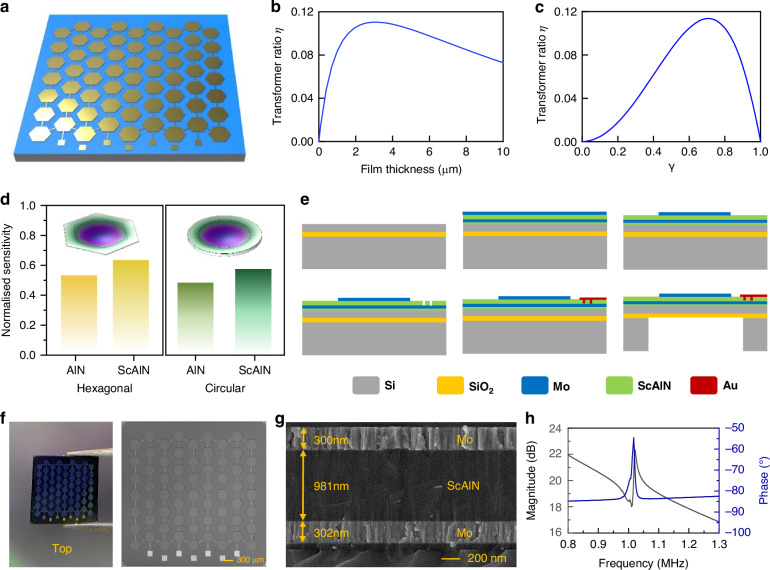
**Design, Manufacturing, and Testing of PMUT Chips**. **a** Schematic diagram of the three-dimensional structure of an acoustic sensor based on SOI chip. Simulation analysis of the influence of piezoelectric film thickness (**b**) and top electrode diameter ratio (**c**) on transmission rate. **d** Simulation analysis of the influence of film shape and material on sensitivity. **e** Detailed fabrication process flow of the PMUTs chips. **f** Front morphology of the chip. **g** SEM image of thin film cross-section. **h** Impedance characteristics of chip

As shown in the Fig. 2a, as the core sensing element of the MEMS AE sensor, the PMUT’s material selection, structural design, and array configuration directly dictate critical performance parameters such as sensitivity and frequency response. Therefore, the transducer chip design is the most crucial phase, fundamentally determining the overall performance of the MEMS AE sensor. The core energy conversion mechanism in conventional bulk piezoelectric transducers relies on the longitudinal piezoelectric coefficient (d_33_), where the direction of the applied electric field remains parallel to the induced strain. In contrast to this mechanism, PMUTs operate based on the transverse piezoelectric coefficient (d_31_)^41^. When the piezoelectric thin film receives external acoustic signals, it undergoes transverse in-plane strain, which directly induces electrical charges on the top and bottom surfaces of the film, thereby accomplishing the acoustic-to-electrical signal conversion^42^. For AE fault monitoring applications, the critical characteristics required of a PMUT are high receiving sensitivity, a broad bandwidth, and excellent in-band flatness.

To develop a high-performance sensing device, we utilized finite element method (FEM) simulations to optimize structural parameters, including the piezoelectric film thickness, electrode coverage ratio, and cavity geometry, as well as the piezoelectric materials (comparing AlN and ScAlN thin films). Given that film thickness and electrode coverage significantly influence the transducer’s conversion efficiency, FEM was employed to determine the optimal parameters to maximize receiving performance. Figure 2b illustrates the output efficiency of varying piezoelectric layer thicknesses on a fixed 5 μm Si support layer, revealing that the film achieves peak output efficiency at a thickness of ~2 μm. For a PMUT with a diameter of 300 μm subjected to a uniform pressure load of 1 Pa, the conversion efficiency curve corresponding to different top electrode diameters is shown in Fig. 2c, indicating that the optimal top electrode diameter is √2/2 times the cavity diameter. Furthermore, we investigated the impact of the PMUT cavity geometry on sensitivity. As shown in Fig. 2d, under identical resonant frequencies, the hexagonal design generally exhibits higher sensitivity compared to square and circular architectures. Additionally, the hexagonal structure offers a superior fill factor than the circular design, thereby enhancing the active area utilization within the array arrangement. Typically, for a given structural design and driving voltage, the receiving sensitivity of a PMUT is proportional to the ratio of the piezoelectric constant to the dielectric constant (d_31_/ε_r_) of the material. Because ScAlN exhibits a higher d_31_/ε_r_ ratio, the ScAlN-based PMUT demonstrates exceptional receiving sensitivity.

To further enhance the receiving sensitivity, multiple PMUT elements were electrically connected in parallel to accumulate more charge during operation. Simultaneously, this array configuration expands the contact area with the target object, enabling the collection of more acoustic signals and thereby improving the overall performance of the AE system. The primary frequency band of AE activities associated with pipeline transmission, such as leakages, cracks, corrosion, and external impacts, ranges from 40 kHz to 600 kHz. To ensure the sensor possesses an excellent sensitivity response while accounting for the impact of packaging, we targeted the unpackaged PMUT’s resonant frequency at 1 MHz via FEM simulations. Following encapsulation with the matching layer, the resonant frequency of the PMUT sensor shifts to ~300 kHz, positioning it squarely in the middle of the designed AE sensor bandwidth. This strategy effectively guarantees that the AE sensor maintains a certain degree of in-band flatness while exhibiting high sensitivity.

Subsequently, we fabricated the ScAlN-based PMUT using standard MEMS processes requiring a four-mask process flow comprising six main steps, as illustrated in Fig. 2e. The fabrication commenced with the cleaning of a Silicon-on-Insulator (SOI) wafer (6N600-1-5N, Okmetic). This SOI wafer consisted of a 5 μm silicon device layer, a 1 μm buried oxide (BOX) layer, and a 400 μm silicon handle substrate. First, an ~100 nm ScAlN seed layer was grown via atomic layer deposition (ALD) (NLD-4000, Nanomaster) to reduce the surface roughness for subsequent thin-film deposition, thereby optimizing the structure and morphology of the Mo electrodes and the c-axis oriented ScAlN film. Subsequently, physical vapor deposition (PVD) (Sigma, SPTS) was utilized to sequentially deposit 0.3 μm Mo, 1 μm ScAlN, and 0.3 μm Mo thin films onto the seed layer. In the subsequent patterning steps, inductively coupled plasma (ICP) etching (GSEC200, NMC) was employed to define the top Mo electrode and to etch through the ScAlN layer to form vias for bottom electrode access. Next, 200 nm of Au was deposited via magnetron sputtering (MS150X-L, FHR) and patterned using a lift-off process to form metallic routing and contact pads, facilitating the electrical parallel connection among the micro-elements. Finally, deep reactive ion etching (DRIE) (Omega LPX Rapier, SPTS) was executed from the backside of the SOI wafer to release the cavity and form the suspended thin-film vibrating structure.

Figure 2f presents the top view and optical microscope images of the PMUT, with the designed sensor array footprint measuring 3.5 mm × 3.5 mm. Figure 2g displays a cross-sectional scanning electron microscope (SEM) image of the fabricated Mo/ScAlN/Mo stack, clearly showing distinct boundaries between the layers. The measured thicknesses of the sensitive membrane layers are 300 nm / 981 nm / 302 nm, exhibiting minimal deviation from the target values. The sputtered ScAlN thin film exhibits excellent crystal orientation. Notably, Molybdenum (Mo) was selected for the top and bottom electrodes due to its excellent lattice matching with ScAlN. Energy-dispersive X-ray spectroscopy (EDS) analysis confirmed the uniform distribution of Sc, Al, and N elements within the piezoelectric ceramic (Supplementary Figure S1). X-ray diffraction (XRD) measurements indicated that the (002) peak of the ScAlN thin film appeared near 36° (Supplementary Fig. S2). The impedance curve of the device was measured using a network analyzer (E5080A, Keysight). Figure 2h demonstrates that the resonant frequency of the fabricated PMUT sensor is 1.047 MHz, which is in close agreement with the simulated target of 1 MHz. Minor discrepancies in the resonant frequency are primarily attributed to dimensional variations during back-cavity etching and residual thin-film stress.

### System integration of the MEMS AE sensor

Acoustic impedance (*Z*), a core parameter characterizing the acoustic properties of a medium, is defined as the product of the medium’s density (*ρ*) and the speed of sound propagating through it (*c*), expressed as Z=*ρc*. Its fundamental physical significance lies in describing the medium’s resistance to acoustic wave propagation, serving as the crucial theoretical basis for determining the energy distribution and transmission efficiency at the interface between different media. The core logic of this theory revolves around regulating acoustic energy transfer through impedance matching. Specifically, the reflection and transmission behaviors, as well as the degree of energy dissipation, at the interface of two media (Supplementary Fig. S3). The energy transmission coefficient (*T*) can be expressed as^43^:

$${T}_{13}=\frac{4{Z}_{1}{Z}_{3}}{{({Z}_{1}+{Z}_{3})}^{2}{\cos }^{2}{K}_{2}{L}_{2}+{({Z}_{2}+{Z}_{1}{Z}_{3}/{Z}_{2})}^{2}{\sin }^{2}{K}_{2}{L}_{2}}$$

where *T* is the transmission coefficient, *Z*_i_ is the acoustic impedance of the respective medium, k_2_ is the wave number in medium 2, and *L*_2_ is the thickness of medium 2.

Acoustic impedance matching is the critical factor dictating the energy transfer efficiency between the sensor and the acoustic transmission medium. Severe impedance mismatch induces significant energy reflection, thereby reducing the transmission coefficient of acoustic energy. Therefore, an appropriate acoustic impedance matching design can effectively enhance the energy transfer efficiency between the AE sensor and the transmission medium. When the thickness of the intermediate matching layer is an odd multiple of a quarter wavelength(${L}_{2}=(2n+1)\lambda /4$), the energy transmission coefficient simplifies to:

$${T}_{13}=\frac{4{Z}_{1}{Z}_{3}}{{({Z}_{2}+{Z}_{1}{Z}_{3}/{Z}_{2})}^{2}}$$

When the acoustic impedance of medium 2 satisfies ${Z}_{2}=\sqrt{{Z}_{1}{Z}_{3}}$, the transmission coefficient *T* approaches 1. This implies that, under ideal conditions without considering intrinsic acoustic attenuation, selecting a matching medium with a specific acoustic impedance and optimal thickness can theoretically eliminate acoustic reflection, achieving 100% transmission. Achieving acoustic impedance matching between the high-impedance metal pipeline and the low-impedance MEMS AE sensor is paramount for ensuring signal transmission efficiency and minimizing energy loss. However, single-phase solid materials inherently suffer from high acoustic impedance, excessive rigidity, and poor flexibility. These characteristics not only impose significant constraints on the impedance matching design for microelectronic structures but also severely compromise interfacial mechanical compatibility. Direct adhesion often triggers issues such as physical scratching of the chip surface, interfacial stress concentration, and microstructural damage. To address this, we propose a technical scheme for constructing a composite matching layer using a low-impedance epoxy resin as the matrix, doped with high-impedance Al_2_O_3_ powder. This strategy enables precise matching to the target acoustic impedance by tuning the mass ratio of the powder to the matrix. Concurrently, it significantly optimizes the mechanical compliance of the matching material, effectively resolving the compatibility dilemma between single-phase materials and delicate microelectronic structures.

**Fig. 3:**
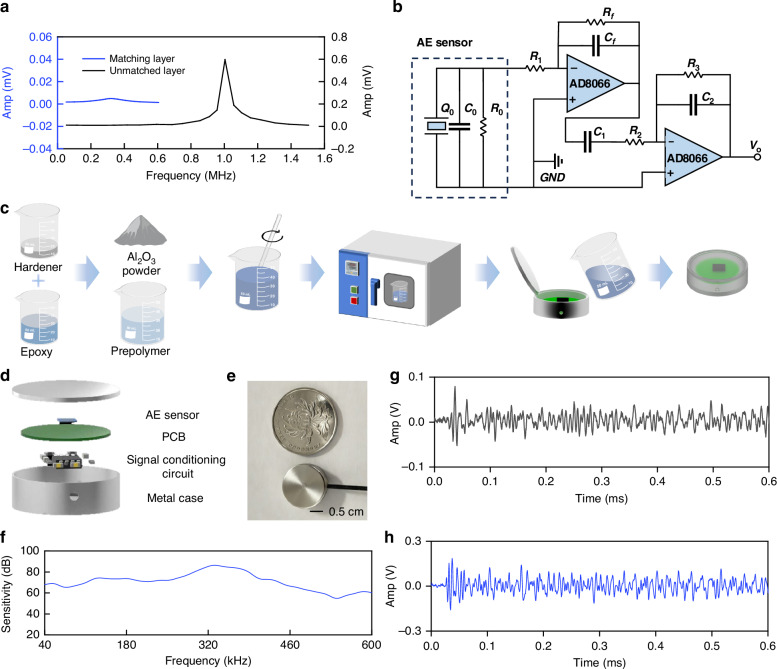
**System Integration and Testing of MEMS AE Sensors**. **a** Frequency response curves of the sensor before and after packaging. **b** Schematic diagram of the signal conditioning circuit. **c** Flowchart of the packaging process. **d** Exploded view illustrating the structural components of the AE sensor. **e** Optical photographs of the fabricated AE sensors. **f** Sensitivity calibration curve of the AE sensor obtained via the comparison method. Pencil lead break test signals captured by (**g**) a traditional AE sensor and (**h**) the proposed MEMS AE sensor

It is noteworthy that the acoustic impedance matching encapsulation structure significantly alters the sensor’s resonant characteristics due to the mass loading effect. Accordingly, we conducted FEM resonance curve simulations. As shown in Fig. 3a, in the unpackaged state, the designed PMUT exhibits a resonant frequency of approximately 1 MHz with a peak output voltage reaching 0.6 mV. Following encapsulation with the matching layer, the resonant frequency shifts to approximately 300 kHz, and the peak output decreases to 0.005 mV. This resonant frequency shift behavior provides a critical theoretical basis for subsequent device optimization. Furthermore, the epoxy-based composite matching layer is inherently viscoelastic, possessing a relatively high mechanical damping coefficient. While the mass loading effect shifts the resonant frequency down to ~300 kHz, the strong damping effect significantly reduces the mechanical quality factor (Q-factor) of the PMUT. For broadband acoustic emission sensing, this reduction in Q-factor is highly desirable. The damping mechanism suppresses the sharp resonant peak and provides mechanical broadening, which intrinsically contributes to the excellent overall bandwidth flatness of the packaged sensor across the 40–600 kHz range. The measured source capacitance (*C*_*0*_ ≈ 330 pF) is sufficiently large to dominate over the unpredictable parasitic capacitances (*C*_*p*_) introduced by the packaging and PCB traces, thereby ensuring stable sensor characteristics. More importantly, the noise gain of a charge amplifier is proportional to 1 + *C*_*0*_/*C*_*f*_. By selecting *C*_*f*_ = 100 pF, the noise gain is constrained to a very stable and low level of approximately 4.3. This ensures that the feedback capacitance is large enough to maintain stable closed-loop operation without severe noise amplification, while still being small enough to deliver a sufficiently high primary voltage output (10 mV/pC) for the subsequent amplification stages. This matching strategy fundamentally optimizes the overall SNR. Figure 3b illustrates the schematic of the designed signal amplification circuit, centered around an AD8066 operational amplifier (Analog Devices), which facilitates voltage gain adjustment and signal preprocessing. The primary charge amplifier module provides a charge-to-voltage conversion gain of 10 mV/pC, while the secondary voltage amplifier stage delivers a fixed gain of 40 dB. The synergy of these two stages ensures the effective extraction of ultra-weak AE signals. In this study, epoxy resin E51 was utilized as the matrix, W93 as the curing agent, and 10 μm Al_2_O_3_ powder as the filler phase to prepare an epoxy-alumina composite system with 60 wt% alumina. The acoustic impedance of alumina powder with doping concentrations ranging from 10 wt% to 60 wt% is shown in supplementary Table 1. Furthermore, when the alumina powder doping concentration reaches 80 wt%, the binder agglomerates, making fine encapsulation of the MEMS chip impossible. Therefore, 60 wt% is the optimal solution that balances improved acoustic impedance with process feasibility. This composite was employed as the acoustic matching layer for the AE sensor. The sensor was housed in a cylindrical aluminum casing. The specific encapsulation process, depicted in Fig. 3c, commenced with mixing the epoxy resin and curing agent at a mass ratio of 5:1. The mixture was thoroughly stirred with a glass rod to form a homogeneous, agglomeration-free colloidal matrix. Subsequently, the pre-ground and refined alumina powder was added in batches under continuous stirring to ensure uniform dispersion. The mixed slurry was then transferred to a vacuum chamber for degassing to eliminate internal voids, thereby enhancing material density and performance stability. Finally, the degassed composite was carefully injected into the aluminum housing and sealed with a precisely fitted metal end cap. The encapsulation was completed by curing the assembly at a constant 50 °C for 1 h or allowing it to cure at room temperature for 24 h. To achieve optimal acoustic impedance matching, enhance structural reliability, and suppress electromagnetic interference, an integrated structural design was adopted for the sensor, as shown in Fig. 3d. From top to bottom, the structure comprises a metal shielding shell, the signal conditioning circuit module, the MEMS AE sensor chip, and a metal encapsulation cover. These layers are tightly bonded to ensure signal transmission stability and robust anti-interference capabilities. Figure 3e provides a size comparison between the packaged sensor and a coin; the final device dimensions are Φ16 mm × h 5.5 mm, weighing only about 3 g, thus exhibiting exceptional miniaturization and lightweight characteristics.

## Results and discussion

### Characterization of the MEMS AE sensor

To systematically characterize the performance of the fabricated MEMS AE sensor, the comparison method, which is currently the standard methodology for calibrating AE sensor sensitivity, was employed, primarily referencing the “JJF 1337-2012 Calibration Specification for Acoustic Emission Sensors (Comparison Method).” The displacement sensitivity of the AE sensor was evaluated under controlled environmental conditions (23 °C, 40% RH). The testing platform consisted of a metal test block, the MEMS AE sensor under test, a standard reference AE sensor, a preamplifier, a pulse signal generator, and a digital oscilloscope (Supplementary Figure S4). As shown in Fig. 3f, the test results demonstrate that the sensor’s sensitivity exceeds 60 dB [ref V/(m/s)] over a broad frequency range from 40 kHz to 600 kHz, with a peak sensitivity of 88.4 dB at 335 kHz, exhibiting excellent broadband and high-sensitivity traits. It should be emphasized that the operating frequency band of the MEMS AE sensor is much wider than the test result, but outside the test frequency band, the sensor’s operating sensitivity is reduced. To further validate the sensor’s capability and reliability in capturing actual AE signals, a Pencil Lead Break (PLB) test (Hsu-Nielsen source) was conducted for AE source simulation. The PLB test is a classical methodology that mimics the transient AE signals generated by actual defect evolution (e.g., crack propagation) using the acoustic wave released upon the fracture of a pencil lead. Due to its stable and reliable signal, broad spectral distribution, simple equipment requirements, and high experimental repeatability, it is widely adopted in laboratory settings. In this experiment, a 2H pencil lead with a diameter of 2.5 mm and an exposed length of 10 mm was utilized. During the test, the angle between the pencil and the metal test block was maintained at 45° (Supplementary Fig. S5). The PLB test results for a commercial AE sensor and the proposed MEMS AE sensor are presented in Fig. 3g and h, respectively. The results indicate that the designed sensor can accurately capture the AE signals generated by the PLB. Furthermore, the signal output amplitude of the proposed sensor is approximately twice that of the commercial AE sensor PXR15, demonstrating a significantly higher SNR.

**Fig. 4:**
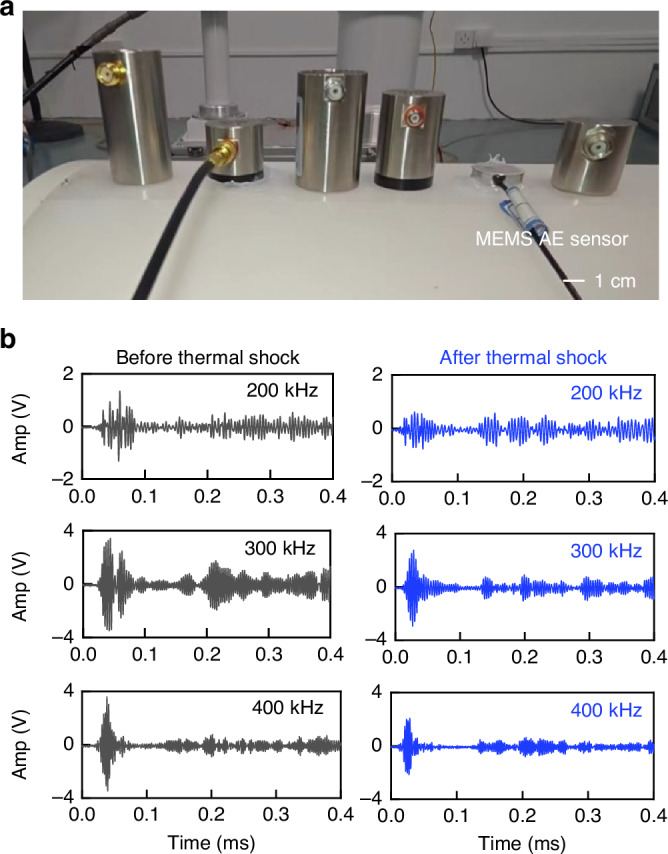
**Physical comparison and thermal reliability testing of the MEMS AE sensor**. **a** Optical photograph illustrating the size comparison between the proposed miniaturized MEMS AE sensor and conventional bulky commercial AE sensors. **b** Time-domain waveform responses of the MEMS AE sensor before (black) and after (blue) thermal shock testing (−55 °C to 85 °C)

To comprehensively and objectively evaluate the overall performance of the developed MEMS AE sensor, several mainstream commercial piezoelectric AE sensors widely utilized in the industry (including PAC R15a, FUJI AE303S, PENGXIANG PXR15, and QINGCHENG GI150/2) were selected as benchmarks. The comparison results of specific core parameters are summarized in Table 1. As indicated by the data, the proposed MEMS AE sensor exhibits a significant generational advantage across all key technical specifications. First, regarding the sensing frequency band, the effective operational bandwidth of conventional commercial devices is typically limited to 50–400 kHz. In contrast, the working bandwidth of the proposed device is substantially broadened to 40–600 kHz, enabling the effective capture of richer, broader-frequency characteristics of early-stage weak structural damage. Second, in terms of peak sensitivity, the core metric dictating the detection limit, the developed device achieves an impressive 88.4 dB. This comprehensively surpasses the best-performing commercial model listed (75 dB), demonstrating an exceptionally strong capability for capturing weak AE events. More outstandingly, benefiting from the inherent advantages of MEMS micro-nanofabrication technology, the physical dimensions of the sensor are remarkably compressed to Φ16 mm × 5.5 mm, with a total weight of merely 3 g. This weight is only one-eighth of that of the lightest commercial model (PXR15, 24 g), completely shattering the technical barriers of the bulky and heavy nature conventionally associated with high-performance piezoelectric transducers. The aforementioned breakthroughs in miniaturization and lightweight design are intuitively validated in the physical comparison shown in Fig. 4a. The figure clearly illustrates the substantial difference in physical profile between the developed MEMS AE sensor and existing large-scale commercial sensors. Traditional commercial AE sensors generally employ bulky, heavy cylindrical metal housings for packaging. This not only occupies significant installation space but also introduces the risk of coupling surface loosening or probe detachment under long-term, high-frequency vibration service conditions due to their substantial self-weight. In contrast, the MEMS sensor developed in this study presents an extremely flattened and lightweight geometric form. The core sensitive film with micrometer-scale thickness, coupled with a highly integrated packaging architecture, not only drastically reduces the material and manufacturing costs of a single device but also enables it to perfectly adapt to constrained application scenarios with extremely strict requirements for installation space and additional weight, such as aerospace engine casings and large-scale complex pipeline networks. Furthermore, its diminutive volume significantly minimizes the mass loading effect on the intrinsic acoustic characteristics of the tested object, laying a solid hardware foundation for realizing ultra-high-density arrayed and imperceptible in-situ online structural health monitoring for critical equipment in the future.

**Table 1:** Performance comparison between the proposed MEMS AE sensor and mainstream commercial AE sensors

Manufacturer	Model	Frequency band(kHz)	Peak sensitivity(dB)	Size (mm)	Weight (g)
PAC	R15a	50–400	62	Φ19×22	32
FUJI	AE303S	30–300	72	Φ20×20	60
PENGXIANG	PXR15	100–400	60	Φ18×18	24
QINGCHENG	GI150/2	60–400	75	Φ30×36	120
This work	/	40–600	88.4	Φ16×5.5	3

The operational environments for AE sensors, such as field transmission pipelines, lathes, and gear reducers, inevitably involve extreme conditions with alternating high and low temperatures. A thermal shock test was conducted to evaluate the performance stability and reliability of the AE sensor under extreme temperature variations. Initially, the AE sensor under test was placed on a metal test block. An emitting transducer serving as the excitation source generated pulsed acoustic signals at various frequencies, and the corresponding responses of the receiving sensor were recorded. Subsequently, the AE sensor was placed into a thermal shock test chamber. The test commenced with the temperature set to the minimum value of −55 °C, where the sensor was held for 2 h to achieve thermal equilibrium. Following this, the ambient temperature was ramped up to 85 °C at a rate of 15 °C/min and maintained for another 2 h, during which the sensor’s performance under rapid temperature fluctuations was monitored. This high-low temperature alternating cycle was repeated 5 times. Upon completion of the cycles, the sensor was removed, and its receiving response at various frequencies was re-tested under identical initial conditions. Figure 4b visually presents the test results at different representative frequencies (200 kHz, 300 kHz, and 400 kHz) before and after the thermal shock testing. The analysis reveals that after multiple −55 °C to 85 °C thermal shock cycles, the key performance metrics of the AE sensor at each characteristic frequency point exhibited no significant attenuation. There were no occurrences of functional degradation, structural damage, or parameter drift. The minor localized variations in the 300 kHz waveform are attributed to the microscopic changes in the acoustic coupling layer and the release of residual packaging stress during thermal cycling, which do not affect the overall sensing reliability. The sensor effectively withstood the aggressive temperature variations from −55 °C to 85 °C without failing, fully validating its excellent thermal shock resistance and confirming its capability to meet the stringent high-reliability requirements of industrial applications.

### Pipeline sabotage monitoring based on the MEMS AE sensor

As core strategic resources within the national energy matrix, the long-distance transmission of oil and natural gas relies heavily on extensive pipeline networks. The secure and stable operation of these networks is directly linked to energy supply security, ecological safety, and public interest. However, during the long-term service of pipelines, human-induced sabotage events (such as illegal oil/gas tapping and unauthorized construction operations) occur frequently. Such incidents not only inflict tremendous economic losses but can also trigger catastrophic safety accidents like leakages, fires, and explosions, posing severe threats to the lives and properties of local residents. The mechanical actions involved in pipeline sabotage, such as digging, striking, and footsteps, are invariably accompanied by characteristic acoustic signals. By capturing and analyzing the time-domain and frequency-domain signatures of these signals, accurate identification and localization of destructive behaviors can be achieved. Therefore, conducting simulated experiments on sabotage behaviors to extract their exclusive acoustic signatures is a critical prerequisite for constructing an efficient pipeline acoustic monitoring system.

**Fig. 5:**
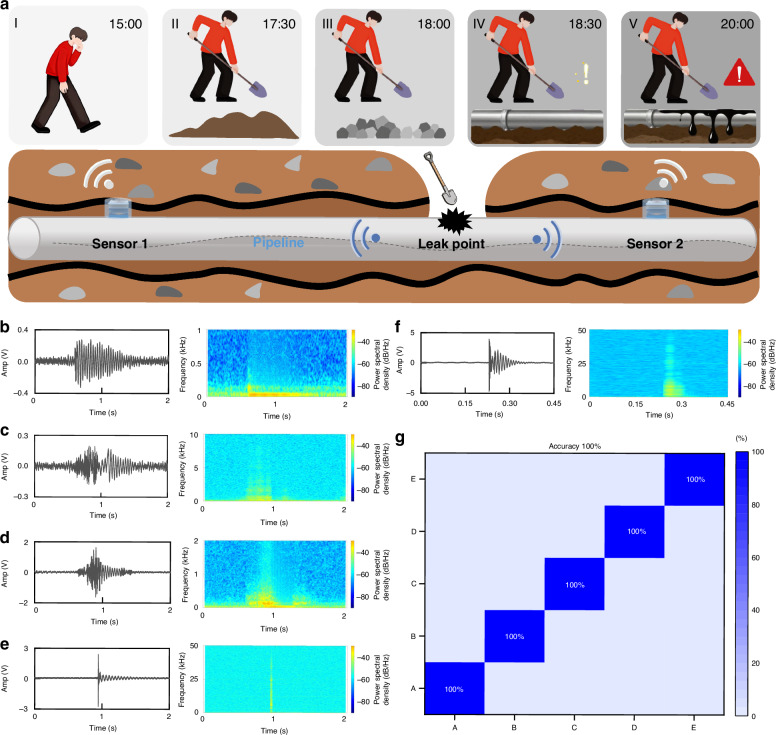
**Application of the MEMS AE sensor in underground pipeline sabotage and leak detection**. **a** Schematic illustration of the simulated pipeline monitoring scenarios, detailing a progression of destructive events (I: footsteps, II: shoveling sand, III: shoveling gravel, IV: tool impact on the pipeline, and V: fluid leakage). **b–f** The captured time-domain acoustic waveforms (left) and their corresponding time-frequency spectrograms (right) for the five distinct events. **g** Confusion matrix of the classification results, demonstrating a 100% recognition accuracy for the five typical pipeline anomaly signals using the deployed pattern recognition algorithm

To systematically investigate the acoustic signal characteristics corresponding to various human-induced sabotage behaviors, a simulated experimental platform comprising a pipeline, sand, gravel, and an acrylic container was constructed to replicate typical field scenarios of pipeline sabotage. Figure 5a chronologically illustrates the complete progression of a simulated sabotage event. Based on this scenario, five core sabotage-related behaviors were selected for focused analysis: the intruder’s footsteps, shoveling sand, shoveling gravel, wooden tool impacts on the pipeline, and metal tool impacts on the pipeline. These lay the experimental foundation for subsequent AE signal feature extraction and pattern recognition. The time-domain waveforms generated by these five behaviors were captured in real-time using an oscilloscope. Subsequently, the raw time-domain signals were subjected to preprocessing, including filtering and denoising, via MATLAB, followed by Fourier transform analysis to obtain the frequency spectra.

Based on the temporal and spectral data, the acoustic features of each core behavior are integrated and analyzed sequentially as follows: Upon the intruder’s arrival, the initial signals generated are footsteps (Fig. 5b). The overall frequency of this acoustic signal is relatively low, maintaining ~100 Hz in the time domain, with a transient peak of 500 Hz occurring exclusively at the moment of foot-to-ground impact. This signature aligns with the impact dynamics and the vibrational response of the ground medium. Subsequently, the sabotage enters the excavation phase with shoveling sand (Fig. 5c). This behavior produces an AE signal with a prolonged duration. A distinct abrupt change appears at the 1 s mark in the time-domain waveform, corresponding to the extraction of the shovel from the sand layer. Frequency-domain analysis reveals that the frictional signal during insertion reaches up to 8 kHz, which is significantly higher than the 2 kHz frequency associated with the extraction motion, establishing a clear differential frequency signature. As the excavation depth increases, the behavior transitions to shoveling gravel (Fig. 5d). While its time-domain characteristics (long duration) are similar to those of shoveling sand, its frequency features differ markedly. The maximum frequency of the signal generated by the collision between the shovel and the gravel is only 1.8 kHz, lower than the frictional frequency of shoveling sand. This discrepancy is directly attributed to the higher density and lower friction coefficient of gravel compared to sand. Upon reaching the pipeline, the critical contact phase begins. The behavior of a wooden tool impacting the pipeline (Fig. 5e) generates an extremely short-duration AE signal characterized by a remarkably high transient frequency peak reaching up to 50 kHz, followed by a prolonged low-frequency tail. In subsequent experiments involving a metal tool impacting the pipeline (Fig. 5f), the core time-frequency characteristics of the AE signal exhibit a high degree of consistency with those of the wooden tool, sharing the common pattern of a short duration and a transient frequency peak of 50 kHz.

In the pattern recognition and feature classification phase, a pre-trained ResNet was introduced as the foundational architecture for transfer learning. This approach leverages universal feature extraction weights learned from massive open-source datasets, thereby accelerating the model’s convergence rate in the target domain and mitigating small-sample overfitting. First, during the data preprocessing stage, the algorithm precisely identified and truncated valid AE signal segments based on predefined amplitude thresholds, partitioning the continuous monitoring data into independent signal samples. Subsequently, the 1D time-series signals acquired by the MEMS AE sensor were converted into 2D time-frequency spectrograms containing rich spatio-temporal information via the Short-Time Fourier Transform (STFT), which served as the input tensors for the deep learning network.

The ResNet architecture constructed in this study comprises 53 convolutional layers and 1 fully connected layer. During the model training process, the 2D time-frequency feature maps underwent iterative computation through convolutional layers, activation functions, and pooling layers. This facilitated highly efficient dimensionality reduction within the feature space and the extraction of deep abstract features. This study contains a total of 1050 time-frequency domain spectrograms, with 210 images for each class. Ultimately, the classification probabilities for the five typical pipeline sabotage behaviors were outputted via the Softmax function in the fully connected layer. In the experiment, the dataset encompassing the five categories of sabotage events was randomly partitioned into a training set (70%) and a validation set (30%) for model evaluation. The testing results demonstrate that the predicted classification outcomes of the trained network model are in profound agreement with the ground-truth labels (see the confusion matrix, Fig. 5g). The convolutional neural network achieved an extraordinary average prediction accuracy of 100% across both the training and validation sets for the five destructive behaviors. These results compellingly demonstrate the tremendous potential of the integrated MEMS AE sensing and deep learning paradigm in the realm of oil and gas pipeline security early warning. It is worth noting that the 100% classification accuracy achieved is primarily attributed to the highly distinct time-frequency signatures of the selected destructive behaviors and the relatively controlled experimental conditions with high SNR. In practical, large-scale deployments, where sensors face complex environmental noise, varying acoustic attenuation, and unpredictable mechanical interferences, this ideal accuracy is expected to experience normal degradation. Future work will focus on expanding the dataset with real-world noisy samples to further evaluate the model’s robustness.

## Conclusions

In this study, we successfully developed a highly integrated, broadband, and high-sensitivity microelectromechanical systems (MEMS) acoustic emission (AE) sensor. Based on a dual-driven paradigm of “hardware sensing + intelligent algorithm,” we constructed an efficient intelligent monitoring system for human-induced pipeline sabotage events. To address the inherent pain points of traditional bulk piezoelectric ceramic AE sensors, such as bulky dimensions, narrow bandwidths, and the inability to achieve low-cost distributed deployment, this research utilized a scandium-doped aluminum nitride (ScAlN) piezoelectric micromachined ultrasonic transducer (PMUT) as the core sensing element. Furthermore, we innovatively engineered a composite matching layer comprising epoxy resin doped with 60 wt% alumina (Al₂O₃) powder. This encapsulation strategy not only achieves highly efficient acoustic impedance matching between the micro-chip and the external medium but also significantly enhances the device’s physical reliability and mechanical compatibility in extreme environments.

System-level performance characterizations reveal that the fabricated MEMS AE sensor maintains a displacement sensitivity exceeding 60 dB across a broad frequency range of 40–600 kHz, achieving a peak sensitivity of 88.4 dB at 335 kHz. In standardized Pencil Lead Break (PLB) tests, its output signal amplitude was approximately twice that of conventional commercial AE sensors, demonstrating an exceptional capability in capturing ultra-weak signals alongside an ultra-high SNR. Moreover, the sensor exhibited absolutely no performance degradation during rigorous thermal shock cycling tests from −55 °C to 85 °C, fully validating its reliability for prolonged service in complex and harsh field industrial environments. Addressing the stringent demands of real-world industrial scenarios, this study further deployed the sensor in a pipeline anti-sabotage early warning application. By constructing a simulated sabotage platform, the exclusive time-frequency characteristics of five core destructive behaviors, including footsteps, shoveling sand, shoveling gravel, and wooden/metal tool impacts on the pipeline, were precisely captured and analyzed. By integrating the STFT with a pre-trained ResNet, we successfully achieved the deep extraction and intelligent classification of high-dimensional AE features. Strikingly, the network model achieved an extraordinary average prediction accuracy of 100% across the five categories of pipeline sabotage behaviors.

In summary, this research comprehensively bridges the entire technological chain of applying micro-acoustic emission technology to pipeline security monitoring, spanning from bottom-layer MEMS chip design and composite acoustic packaging optimization to top-layer deep learning algorithm architectures. The outcomes of this study not only provide a low-cost, high-performance miniaturized hardware platform for the precise early identification and intelligent screening of human-induced pipeline sabotage events, but also offer robust technical support for safeguarding the absolute security of national energy arteries. Concurrently, it profoundly broadens the application prospects of high-performance MEMS AE sensors in the broader realm of Industrial Internet of Things (IIoT) structural health monitoring. While the miniaturization and batch-fabrication capabilities of the proposed MEMS AE sensors demonstrate tremendous potential for high-density, low-cost distributed deployment along vast pipeline networks, realizing large-scale industrial applications still faces practical challenges. Future research efforts should focus on optimizing ultra-low-power edge computing architectures, integrating self-powered technologies, and developing robust multi-node wireless communication protocols to efficiently handle and fuse massive acoustic data in remote environments.

## Supplementary information

Supporting Information_for Production
