# Supplementary material for: Intelligent monitoring system for pipeline status based on MEMS acoustic emission sensors
Source: Microsyst Nanoeng. 2026 Jul 9;12:260. doi: 10.1038/s41378-026-01367-1 (PMC13351072; doi:10.1038/s41378-026-01367-1)
Supplement: Supplementary file 1 — Supporting Information_for Production [file 41378_2026_1367_MOESM1_ESM.docx]

Supplementary Information

**Intelligent monitoring system for pipeline status based on MEMS acoustic emission sensors**

Tao Liu†*, Wenwu Yan†, Dongxiao Li†, Jidong Cai, Jian Feng, Hanjie Dou, Jiaqian Yang, Pengfan Wu, Hongliang Wang and Xiaojing Mu*

**Author Information**

Affiliations

T. Liu, W. Yan, J. Cai, J. Feng, Prof. H. Wang, Science and Technology on Electronic Test and Measurement Laboratory, State key Laboratory of Extreme Environment Optoelectronic Dynamic Measurement Technology and Instrument, North University of China, Taiyuan, 030051, P.R. China

E-mail: liutao@nuc.edu.cn

D. Li, H. Dou, J. Yang, P. Wu, Prof. X. Mu, Key Laboratory of Optoelectronic Technology & Systems Ministry of Education, International R & D center of Micro-nano Systems and New Materials Technology, Chongqing University, Chongqing, 400044, P.R. China

E-mail: mxjacj@cqu.edu.cn

All authors

E-mail: 20250112@nuc.edu.cn; 18335737700@163.com; [lidongxiao@cqu.edu.cn](mailto:lidongxiao@cqu.edu.cn); 18334940084@163.com; [15735601916@163.com](mailto:15735601916@163.com); hanjiedou@cqu.edu.cn; jiaqianyang@stu.cqu.edu.cn; [pengfanwu@cqu.edu.cn](mailto:pengfanwu@cqu.edu.cn); wanghongliang@nuc.edu.cn; mxjacj@cqu.edu.cn.

Corresponding author

E-mail: 20250112@nuc.edu.cn; mxjacj@cqu.edu.cn.

Address: School of Optoelectronic Engineering, Chongqing University, Chongqing 400044, China

† These authors contributed equally to this work

**Supplementary Fig. S1** Grooves about 20 μm wide x 20 μm long x 10 μm deep were opened on the surface of ScAlN thin films using a focused ion beam (FIB, Scios dual-beam scanning electron microscopy /FIB system). The scandium concentration on ScAlN cross section was measured by energy dispersive X-ray spectrometer at 15 keV beam energy. EDX scanning data is shown in the figure. The results show that the scandium concentration remains consistent throughout the film thickness. a corresponding EDX mappings of four key elements for observing ScAlN layers. b EDX results over the line scan.

**Supplementary Fig. S2 The crystal structure of ScAlN was studied by X-ray diffraction (XRD).** a shows the XRD peak of ScAlN thin film on a Mo electrode with a thickness of 1 μm. The swing curve of the peak ScAlN (002) is also measured, as shown in b. The FWHM of ScAlN film is 1.9°, indicating that the c axis of ScAlN film is well arranged, indicating good piezoelectric properties.

**Supplementary Fig.3 Impedance matching principle**.

**Supplementary Table 1 Sample parameters of doped alμmina powder**

| quality score (%) | | 10 | 20 | 30 | 40 | 50 | 60 |
| --- | --- | --- | --- | --- | --- | --- | --- |
| Density *ρ*(kg/m^3^) | 1200 | | 1296 | 1393 | 1522 | 1749 | 1975 |
| Speed of sound *c*(m/s) | 2595 | | 2691 | 2709 | 2735 | 2919 | 2925 |
| Acoustic impedance *Z*(MRayl) | 3.11 | | 3.48 | 3.77 | 4.16 | 5.10 | 5.77 |

**Supplementary Fig.4 Acoustic emission sensor calibration platform.**

**Supplementary Fig.5 Pencil Lead Break (PLB) test platform.**
